# Methyl 3-phenyl­isoxazole-5-carboxyl­ate

**DOI:** 10.1107/S1600536814000038

**Published:** 2014-02-05

**Authors:** Li Wang, Ya-jun Li, Yao-dong Li, Wei Zhang, Rui Xu

**Affiliations:** a Affiliated Hospital of Xi’an Medical College, 48 Fenghao West Road, 710077, Xi’an, People’s Republic of China

## Abstract

In the title compound, C_11_H_9_NO_3_, the dihedral angle between the isoxazole and phenyl rings is 19.79 (12), while the ester group is inclined to the isoxazole group by 12.14 (6)°. In the crystal, mol­ecules are linked by C—H⋯O hydrogen bonds, forming layers lying parallel to (010).

## Related literature   

For the biological activity of isoxazole derivatives, see: Musad *et al.* (2011 ▶). For the synthesis and the structure of a related compound, see: Wang *et al.* (2013 ▶).
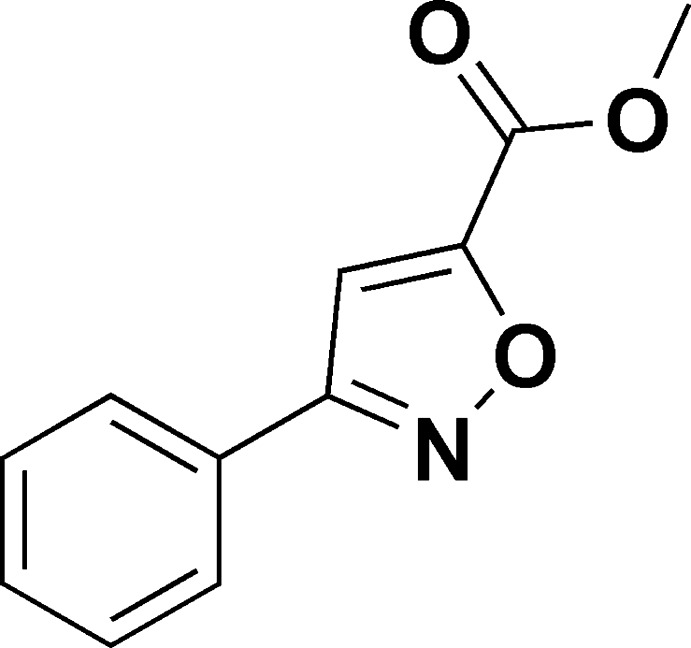


## Experimental   

### 

#### Crystal data  

C_11_H_9_NO_3_
*M* *_r_* = 203.19
Monoclinic, 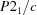

*a* = 12.2275 (18) Å
*b* = 13.604 (2) Å
*c* = 5.8746 (9) Å
β = 97.011 (3)°
*V* = 969.9 (3) Å^3^
*Z* = 4
Mo *K*α radiation
μ = 0.10 mm^−1^
*T* = 296 K
0.36 × 0.25 × 0.13 mm

#### Data collection  

Bruker APEXII CCD diffractometer
Absorption correction: multi-scan (*SADABS*; Bruker, 2008 ▶) *T*_min_ = 0.964, *T*_max_ = 0.987
4807 measured reflections
1718 independent reflections
1238 reflections with *I* > 2σ(*I*)
*R*_int_ = 0.036

#### Refinement  

*R*[*F* ^2^ > 2σ(*F* ^2^)] = 0.059
*wR*(*F* ^2^) = 0.133
*S* = 1.13
1718 reflections
138 parameters
H-atom parameters constrained
Δρ_max_ = 0.17 e Å^−3^
Δρ_min_ = −0.15 e Å^−3^

### 

Data collection: *APEX2* (Bruker, 2008 ▶); cell refinement: *SAINT* (Bruker, 2008 ▶); data reduction: *SAINT*; program(s) used to solve structure: *SHELXS97* (Sheldrick, 2008 ▶); program(s) used to refine structure: *SHELXL97* (Sheldrick, 2008 ▶); molecular graphics: *SHELXTL* (Sheldrick, 2008 ▶); software used to prepare material for publication: *SHELXL97*, *PLATON* (Spek, 2009 ▶) and *publCIF* (Westrip, 2010 ▶).

## Supplementary Material

Crystal structure: contains datablock(s) I, global. DOI: 10.1107/S1600536814000038/su2682sup1.cif

Structure factors: contains datablock(s) I. DOI: 10.1107/S1600536814000038/su2682Isup2.hkl

Supporting information file. DOI: 10.1107/S1600536814000038/su2682Isup3.cml

CCDC reference:

Additional supporting information:  crystallographic information; 3D view; checkCIF report

## Figures and Tables

**Table 1:** Hydrogen-bond geometry (Å, °)

*D*—H⋯*A*	*D*—H	H⋯*A*	*D*⋯*A*	*D*—H⋯*A*
C3—H3⋯O2^i^	0.93	2.58	3.512 (3)	175
C12—H12*B*⋯O2^ii^	0.96	2.50	3.412 (3)	159

Symmetry codes: (i) 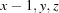; (ii) 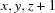.

**Fig. 1.**
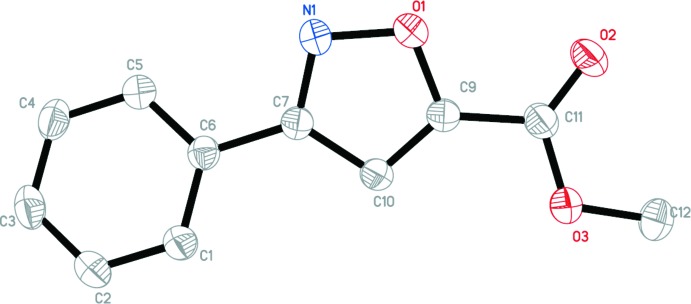
The molecular structure of the title molecule, with atom labelling. Displacement ellipsoids are drawn at the 30% probability level. H atoms have been omitted for clarity.

## supplementary crystallographic information

### 1. Comment

The wide occurrence of heterocycles, such as isoxazoles, in bioactive natural products, pharmaceuticals and agrochemicals has made them important synthetic targets. They are of great importance in biological chemistry, showing anticancer activity, and substituted isoxazoles have revealed antibacterial, antioxidant, insecticidal properties (Musad *et al.*, 2011). Here we report on the crystal structure of the title isoxazole derivative, synthesized by alcoholysis of 3-Phenyl-isoxazole-5-carbonyl chloride in dichloromethane.

In the molecule of the title compound, Fig. 1, the dihedral angle between the phenyl and the isoxazole rings is 19.79 (12) °. This is larger than that of 7.37 (19)° observed in the related compound Isopropyl 3-phenylisoxazole-5-carboxylate (Wang *et al.*, 2013), but the bond lengths within the isoxazole ring are the same.

In the crystal, molecules are linked by C—H···O hydrogen bonds (Table 1), forming layers lying parallel to (010).

### 2. Experimental

3-Phenylisoxazole-5-carboxylic acid (10 mmol, 1.95 g; Wang *et al.*, 2013) was dissolved in 100 ml dichloromethane, then thionyl chloride (12 mmol, 1.43 g) was added drop wise while the solution was stirred for 20 minutes in an ice bath. The solvent was removed under reduced pressure and the mixture was used for the next step without further purification. Methanol (20 mmol, 0.64 g) was then added and the mixture stirred for 6 h at room temperature. The resulting residue was purified as a white solid (1.54 g; 76% yield). Recrystallization in dichloromethane gave fine colourless plate-like crystals suitable for X-ray diffraction analysis.

### 3. Refinement

All H atoms were placed in idealized positions and allowed to ride on the respective parent atom: C—H = 0.93–0.96 Å with *U*_iso_(H) = 1.5*U*_eq_(*C*-methyl) and = 1.2*U*_eq_(C) for other H atoms.

### Crystal data

**Table:**

C_11_H_9_NO_3_	*Z* = 4
*M**_r_* = 203.19	*F*(000) = 424
Monoclinic, *P*2_1_/*c*	*D*_x_ = 1.392 Mg m^−^^3^
Hall symbol: -P 2ybc	Mo *K*α radiation, λ = 0.71073 Å
*a* = 12.2275 (18) Å	θ = 1.7–25.1°
*b* = 13.604 (2) Å	µ = 0.10 mm^−^^1^
*c* = 5.8746 (9) Å	*T* = 296 K
β = 97.011 (3)°	Plate, colourless
*V* = 969.9 (3) Å^3^	0.36 × 0.25 × 0.13 mm

### Data collection

**Table:**

Bruker APEXII CCD diffractometer	1718 independent reflections
Radiation source: fine-focus sealed tube	1238 reflections with *I* > 2σ(*I*)
Graphite monochromator	*R*_int_ = 0.036
phi and ω scans	θ_max_ = 25.1°, θ_min_ = 1.7°
Absorption correction: multi-scan (*SADABS*; Bruker, 2008)	*h* = −14→10
*T*_min_ = 0.964, *T*_max_ = 0.987	*k* = −15→16
4807 measured reflections	*l* = −6→7

### Refinement

**Table:**

Refinement on *F*^2^	Secondary atom site location: difference Fourier map
Least-squares matrix: full	Hydrogen site location: inferred from neighbouring sites
*R*[*F*^2^ > 2σ(*F*^2^)] = 0.059	H-atom parameters constrained
*wR*(*F*^2^) = 0.133	*w* = 1/[σ^2^(*F*_o_^2^) + (0.0531*P*)^2^] where *P* = (*F*_o_^2^ + 2*F*_c_^2^)/3
*S* = 1.13	(Δ/σ)_max_ < 0.001
1718 reflections	Δρ_max_ = 0.17 e Å^−^^3^
138 parameters	Δρ_min_ = −0.15 e Å^−^^3^
0 restraints	Extinction correction: *SHELXL*, Fc^*^=kFc[1+0.001xFc^2^λ^3^/sin(2θ)]^-1/4^
Primary atom site location: structure-invariant direct methods	Extinction coefficient: 0.0049 (19)

### Special details

**Table:**

Geometry. All e.s.d.'s (except the e.s.d. in the dihedral angle between two l.s. planes) are estimated using the full covariance matrix. The cell e.s.d.'s are taken into account individually in the estimation of e.s.d.'s in distances, angles and torsion angles; correlations between e.s.d.'s in cell parameters are only used when they are defined by crystal symmetry. An approximate (isotropic) treatment of cell e.s.d.'s is used for estimating e.s.d.'s involving l.s. planes.
Refinement. Refinement of *F*^2^ against ALL reflections. The weighted *R*-factor *wR* and goodness of fit *S* are based on *F*^2^, conventional *R*-factors *R* are based on *F*, with *F* set to zero for negative *F*^2^. The threshold expression of *F*^2^ > σ(*F*^2^) is used only for calculating *R*-factors(gt) *etc*. and is not relevant to the choice of reflections for refinement. *R*-factors based on *F*^2^ are statistically about twice as large as those based on *F*, and *R*-factors based on ALL data will be even larger.

### Fractional atomic coordinates and isotropic or equivalent isotropic displacement parameters (Å^2^)

**Table:**

	*x*	*y*	*z*	*U*_iso_*/*U*_eq_	
N1	0.58165 (16)	0.12445 (15)	0.1263 (4)	0.0548 (6)	
O1	0.69685 (13)	0.12779 (12)	0.1714 (3)	0.0568 (5)	
O2	0.91297 (15)	0.11346 (15)	0.3629 (3)	0.0734 (7)	
O3	0.85964 (13)	0.14679 (13)	0.7065 (3)	0.0601 (5)	
C1	0.3828 (2)	0.15075 (17)	0.5374 (4)	0.0497 (7)	
H1	0.4298	0.1742	0.6620	0.060*	
C2	0.2707 (2)	0.14652 (19)	0.5489 (5)	0.0581 (7)	
H2	0.2426	0.1677	0.6807	0.070*	
C3	0.2004 (2)	0.11121 (19)	0.3669 (5)	0.0593 (8)	
H3	0.1249	0.1088	0.3746	0.071*	
C4	0.2428 (2)	0.07947 (19)	0.1727 (5)	0.0585 (7)	
H4	0.1956	0.0546	0.0501	0.070*	
C5	0.35431 (19)	0.08422 (18)	0.1585 (4)	0.0495 (7)	
H5	0.3820	0.0632	0.0261	0.059*	
C6	0.42544 (18)	0.12036 (15)	0.3418 (4)	0.0419 (6)	
C7	0.54474 (18)	0.12641 (15)	0.3261 (4)	0.0404 (6)	
C9	0.72372 (19)	0.13278 (17)	0.4011 (4)	0.0447 (6)	
C10	0.63299 (18)	0.13246 (16)	0.5065 (4)	0.0451 (6)	
H10	0.6286	0.1355	0.6633	0.054*	
C11	0.8426 (2)	0.13030 (18)	0.4824 (5)	0.0511 (7)	
C12	0.9720 (2)	0.1372 (2)	0.8127 (5)	0.0722 (9)	
H12A	1.0015	0.0755	0.7694	0.108*	
H12B	0.9737	0.1397	0.9764	0.108*	
H12C	1.0154	0.1900	0.7629	0.108*	

### Atomic displacement parameters (Å^2^)

**Table:**

	*U*^11^	*U*^22^	*U*^33^	*U*^12^	*U*^13^	*U*^23^
N1	0.0377 (12)	0.0773 (16)	0.0498 (14)	−0.0009 (10)	0.0068 (10)	−0.0028 (11)
O1	0.0444 (11)	0.0800 (13)	0.0475 (11)	−0.0009 (9)	0.0119 (8)	−0.0010 (9)
O2	0.0451 (11)	0.1176 (18)	0.0603 (13)	0.0086 (11)	0.0184 (9)	0.0003 (11)
O3	0.0400 (10)	0.0877 (14)	0.0523 (12)	0.0010 (9)	0.0044 (8)	−0.0068 (10)
C1	0.0448 (15)	0.0555 (16)	0.0492 (16)	−0.0026 (12)	0.0074 (12)	−0.0066 (12)
C2	0.0490 (17)	0.0677 (18)	0.0603 (18)	0.0044 (13)	0.0173 (13)	−0.0047 (14)
C3	0.0363 (14)	0.0689 (19)	0.073 (2)	0.0013 (13)	0.0078 (14)	0.0140 (15)
C4	0.0425 (16)	0.0702 (18)	0.0598 (19)	−0.0053 (13)	−0.0062 (13)	0.0057 (14)
C5	0.0455 (15)	0.0563 (15)	0.0466 (16)	0.0009 (12)	0.0054 (12)	−0.0007 (12)
C6	0.0406 (14)	0.0411 (13)	0.0438 (14)	0.0013 (10)	0.0039 (11)	0.0035 (11)
C7	0.0412 (14)	0.0371 (13)	0.0427 (14)	0.0005 (10)	0.0040 (11)	−0.0020 (11)
C9	0.0441 (15)	0.0497 (15)	0.0405 (14)	−0.0008 (11)	0.0057 (11)	0.0024 (11)
C10	0.0427 (14)	0.0525 (15)	0.0407 (14)	0.0043 (12)	0.0075 (11)	−0.0007 (11)
C11	0.0451 (16)	0.0565 (16)	0.0529 (17)	0.0014 (12)	0.0108 (13)	0.0010 (13)
C12	0.0457 (17)	0.104 (2)	0.0639 (19)	0.0039 (15)	−0.0067 (14)	−0.0015 (17)

### Geometric parameters (Å, º)

**Table:**

N1—C7	1.308 (3)	C4—C5	1.378 (3)
N1—O1	1.402 (2)	C4—H4	0.9300
O1—C9	1.351 (3)	C5—C6	1.389 (3)
O2—C11	1.197 (3)	C5—H5	0.9300
O3—C11	1.326 (3)	C6—C7	1.475 (3)
O3—C12	1.443 (3)	C7—C10	1.420 (3)
C1—C6	1.382 (3)	C9—C10	1.335 (3)
C1—C2	1.382 (3)	C9—C11	1.474 (3)
C1—H1	0.9300	C10—H10	0.9300
C2—C3	1.374 (4)	C12—H12A	0.9600
C2—H2	0.9300	C12—H12B	0.9600
C3—C4	1.379 (4)	C12—H12C	0.9600
C3—H3	0.9300		
			
C7—N1—O1	106.16 (18)	C5—C6—C7	120.1 (2)
C9—O1—N1	107.86 (17)	N1—C7—C10	110.9 (2)
C11—O3—C12	116.1 (2)	N1—C7—C6	120.5 (2)
C6—C1—C2	120.3 (2)	C10—C7—C6	128.6 (2)
C6—C1—H1	119.8	C10—C9—O1	110.4 (2)
C2—C1—H1	119.8	C10—C9—C11	133.8 (2)
C3—C2—C1	120.4 (3)	O1—C9—C11	115.6 (2)
C3—C2—H2	119.8	C9—C10—C7	104.6 (2)
C1—C2—H2	119.8	C9—C10—H10	127.7
C2—C3—C4	119.4 (2)	C7—C10—H10	127.7
C2—C3—H3	120.3	O2—C11—O3	125.2 (2)
C4—C3—H3	120.3	O2—C11—C9	124.4 (3)
C5—C4—C3	120.7 (2)	O3—C11—C9	110.3 (2)
C5—C4—H4	119.7	O3—C12—H12A	109.5
C3—C4—H4	119.7	O3—C12—H12B	109.5
C4—C5—C6	120.0 (2)	H12A—C12—H12B	109.5
C4—C5—H5	120.0	O3—C12—H12C	109.5
C6—C5—H5	120.0	H12A—C12—H12C	109.5
C1—C6—C5	119.1 (2)	H12B—C12—H12C	109.5
C1—C6—C7	120.8 (2)		
			
C7—N1—O1—C9	−0.7 (3)	C5—C6—C7—C10	−159.5 (2)
C6—C1—C2—C3	0.6 (4)	N1—O1—C9—C10	0.2 (3)
C1—C2—C3—C4	0.3 (4)	N1—O1—C9—C11	176.1 (2)
C2—C3—C4—C5	−1.0 (4)	O1—C9—C10—C7	0.2 (3)
C3—C4—C5—C6	0.7 (4)	C11—C9—C10—C7	−174.6 (3)
C2—C1—C6—C5	−0.9 (3)	N1—C7—C10—C9	−0.7 (3)
C2—C1—C6—C7	178.8 (2)	C6—C7—C10—C9	177.9 (2)
C4—C5—C6—C1	0.3 (3)	C12—O3—C11—O2	−4.0 (4)
C4—C5—C6—C7	−179.4 (2)	C12—O3—C11—C9	174.4 (2)
O1—N1—C7—C10	0.8 (3)	C10—C9—C11—O2	166.0 (3)
O1—N1—C7—C6	−177.87 (18)	O1—C9—C11—O2	−8.7 (4)
C1—C6—C7—N1	−160.8 (2)	C10—C9—C11—O3	−12.4 (4)
C5—C6—C7—N1	18.9 (3)	O1—C9—C11—O3	172.9 (2)
C1—C6—C7—C10	20.8 (4)		

### Hydrogen-bond geometry (Å, º)

**Table:**

*D*—H···*A*	*D*—H	H···*A*	*D*···*A*	*D*—H···*A*
C3—H3···O2^i^	0.93	2.58	3.512 (3)	175
C12—H12*B*···O2^ii^	0.96	2.50	3.412 (3)	159

Symmetry codes: (i) *x*−1, *y*, *z*; (ii) *x*, *y*, *z*+1.
